# Angiogenesis in a human neuroblastoma xenograft model: mechanisms and inhibition by tumour-derived interferon-*γ*

**DOI:** 10.1038/sj.bjc.6603186

**Published:** 2006-05-23

**Authors:** D Ribatti, B Nico, A Pezzolo, A Vacca, R Meazza, R Cinti, B Carlini, F Parodi, V Pistoia, M V Corrias

**Affiliations:** 1 Department of Human Anatomy and Histology, University of Bari Medical School, Bari, Italy; 2 Laboratory of Oncology, Gaslini Institute, Genoa, Italy; 3 Department of Internal Medicine and Clinical Oncology, University of Bari Medical School, Bari, Italy; 4 Laboratory of Clinical and Experimental Immunology, Genoa, Italy; 5 Service of Pathology, Gaslini Institute, Genoa, Italy

**Keywords:** interferon-*γ*, angiostatic, neuroblastoma, tumour, endothelial cells

## Abstract

Tumour progression in neuroblastoma (NB) patients correlates with high vascular index. We have previously shown that the ACN NB cell line is tumorigenic and angiogenic in immunodeficient mice, and that interferon-*γ* (IFN-*γ*) gene transfer dampens ACN tumorigenicity. As IFN-*γ* represses lymphocyte-induced tumour angiogenesis in various murine models and inhibits proliferation and migration of human endothelial cells, we have investigated the antiangiogenic activity of tumour-derived IFN-*γ* and the underlying mechanism(s). In addition, we characterised the tumour vasculature of the ACN xenografts, using the chick embryo chorioallantoic membrane assay. We show that the *ACN/IFN-γ* xenografts had a lower microvessel density and less *in vivo* angiogenic potential than the vector-transfected *ACN/neo*. The vascular channels of both xenografts were formed by a mixed endothelial cell population of murine and human origin, as assessed by the FICTION (fluorescence immunophenotyping and interphase cytogenetics) technique. With respect to *ACN/neo,* the *ACN/IFN-γ* xenografts showed more terminal deoxynucleotidyl transferase-mediated dUTP nick end labelling-positive human and murine endothelial cells, suggesting that inhibition of angiogenesis by IFN-*γ* was dependent on the induction of apoptosis, likely mediated by nitric oxide. Once the dual origin of tumour vasculature is confirmed in NB patients, the xenograft model described here will prove useful in testing the efficacy of different antiangiogenic compounds.

Neuroblastoma (NB) is the most common malignant tumour in infants and the fourth most common malignancy in children older than 1 year of age (Brodeur and Maris, 2001). Neuroblastoma may regress spontaneously in infants, mature to benign ganglioneuromas in older children, or grow relentlessly and be rapidly fatal (Brodeur and Maris, 2001). In NB, angiogenesis appears to play an important role in determining tumour phenotype (Ribatti *et al*, 2004). A spectrum of angiogenesis stimulators, such as vascular endothelial growth factor and fibroblast growth factor-2 (FGF-2), as well as inhibitors, such as tissue inhibitors of matrix metalloproteinases (MMP), have been detected in NB tumours (Ara *et al*, 1998; Meister *et al*, 1999; Eggert *et al*, 2000). Moreover, increased production of MMP-2 and -9 has been observed in advanced disease stages, favouring degradation of extracellular matrix and enhancing tumour dissemination (Ara *et al*, 1998; Sugiura *et al*, 1998; Sakakibara *et al*, 1999; Ribatti *et al*, 2001b). High tumour vascularity correlates with metastatic disease, MYC amplification, unfavourable histology and poor outcome; by contrast, low tumour vascularity is associated with favourable prognostic features, such as localised disease and favourable histology (Meitar *et al*, 1996; Canete *et al*, 2000; Katzenstein *et al*, 2000; Ribatti *et al*, 2002).

Thus, inhibition of angiogenesis may represent a useful approach for adjuvant therapy (Ribatti and Ponzoni, 2005) in metastatic NB patients, whose survival is poor (Maris and Matthay, 1999). However, evaluation of efficacy of potential angiogenic inhibitors requires a better understanding of the *in vivo* angiogenic potential of NB cells and of their contribution to vasculogenic mimicry (Folberg and Mantiotis, 2004). Endothelial cells showing the same genetic alteration as tumour cells have recently been found in human tumours (Gunsilius, 2003; Hida *et al*, 2004; Streubel *et al*, 2004), supporting the hypothesis that this could be a general phenomenon.

To address these questions, we took advantage of the highly angiogenic phenotype of the human NB cell line ACN in immunodeficient mice (Corrias *et al*, 1998). Parental and vector-transfected ACN xenografts showed numerous blood vessels and a well-defined vascularisation in the small fibrous bands lining the tumour cell nests (Corrias *et al*, 1998; Airoldi *et al*, 2004). By contrast, human interferon-*γ* (IFN-*γ*) transfectans showed focal basement membrane destruction and alterations in the microvascular architecture, thereby supporting the hypothesis that IFN-*γ* could affect the angiogenic potential of NB cells besides reducing tumour cell proliferation (Airoldi *et al*, 2004). Antiangiogenic effects of IFN-*γ*, in fact, have been described in humans as well as in several *in vitro* and *in vivo* models. Precisely, murine IFN-*γ* produced by either CD4+ or CD8+ cells inhibits tumour-induced angiogenesis in syngeneic tumour models (Saiki *et al*, 1992; Qin and Blankenstein, 2000; Blankenstein and Qin, 2003). Human IFN-*γ* inhibits proliferation and migration of human endothelial cells and capillary tube formation *in vitro* (Brouty-Boye and Zetter, 1980; Friesel *et al*, 1987; Tsuruoka *et al*, 1988; Maheshwari *et al*, 1991; Albini *et al*, 2000) and represses lymphocyte-induced tumour angiogenesis (Sidky and Borden, 1987).

Here, using the chick embryo chorioallantoic membrane (CAM) assay (Ribatti *et al*, 2001a), we show that the *ACN/IFN-γ* xenografts have a lower microvessel density and decreased angiogenic potential *in vivo* compared to vector-transfected *ACN/neo* cells. Moreover, the antiangiogenic activity of *ACN/IFN-γ* xenografts affected vascular channels by increasing apoptosis of both murine and human endothelial cells, likely through nitric oxide (NO) production.

## MATERIALS AND METHODS

### Human IFN-*γ*-transfected cell line

The NB cell line ACN was stably transfected with a human IFN-*γ* cDNA cloned in the *Xba*I blunted-*Bam*HI sites of plasmid RSV.5 neo (later referred as *ACN/IFN-γ*) or with the empty vector (later referred as *ACN/neo*) (Airoldi *et al*, 2004).

### Nude mice studies

Pathogen-free female athymic (nu/nu) mice, 6–8 weeks old, were obtained from Harlan Italy (San Pietro al Natisone, Italy). Animal experiments, performed according to the National Regulation on Animal Research, were approved by the Review Board of the Istituto Nazionale per la Ricerca sul Cancro (Genoa, Italy). Mice were housed under sterile conditions and received autoclaved food and water. Animals (10 for each group) were injected subcutaneously with 2 × 10^7^ *ACN/neo* or *ACN/IFN-γ* cells. Tumours were removed on days 8, 14 and 28 post injection (p.i.) and fixed either in 4% paraformaldehyde or Bouin's solution. For CAM experiments, tumours were removed on day 28 and snap-frozen in liquid nitrogen and stored at −80°C until tests were performed.

### Immunohistochemical study

After fixation, *ACN/neo* and *ACN/IFN-γ* tumours were embedded in paraffin, sectioned at 4 *μ*m and stained with haematoxylin–eosin for histological evaluation. For immunohistochemistry, a murine monoclonal antibody (mAb) against murine CD31 (clone 1A10, Dako, Glostrup, Denmark), a more sensitive endothelial cell marker than factor VIII antigen (Horak *et al*, 1992), was used. Briefly, sections were collected on 3-amino-propyl-triethoxysilane-coated slides, deparaffinised by the xylene–ethanol sequence, rehydrated in a graded ethanol scale and in Tris-buffered saline (TBS, pH 7.6) and incubated overnight at 4°C with the mAb 1A10 (1 : 25 in TBS), after antigen retrieval by enzymatic digestion with Ficin (Sigma, St Louis, MO, USA) for 30 min at room temperature. Immunodetection was performed with alkaline phosphatase anti-alkaline phosphatase (Dako) and Fast Red as chromogen, followed by haematoxylin counterstaining. Negative control was an unrelated monoclonal IgG1 produced by the P3X63/Ag8 mouse secretory myeloma (Vacca *et al*, 1993). For CXCL10 and CXCL9 expression studies, sections were incubated with an anti-human CXCL10 (IP10) mAb (ab8098, Abcam Ltd, Cambridge, UK) and an anti-human CXCL9 (Mig) polyclonal antibody (500-P50, Tebu-Bio, Combs, UK) and developed as above.

### Determination of microvessel area

These experiments were simultaneously and independently performed by two investigators (DR and BN) using a computerised image analysis system (Leica Quantimet 5000, Wetzlar, Germany). Four to six × 250 fields, covering almost entirely each of the three sections tested (the third section of a series of three sections each for a total of nine serial sections), per sample were examined with a 144-intersection-point square reticulum (0.0186 mm^2^ field^−1^ and 129.13 *μ*m^2^ point^−1^) inserted in the eyepiece. Care was taken to select microvessels, that is, capillaries and small venules, from all the CD31-stained vessels. These vessels were identified as transversally sectioned tubes with a single layer of endothelial cells, without or with a lumen (diameter ranging from 3 to 10 *μ*m). Each assessment was agreed upon in turn. Microvessels were counted by a planimetric ‘point-count method’ (Elias and Hyde, 1983) with slight modifications, whereby only transversally cut microvessels occupying the reticulum points were counted. As the microvessel diameter was smaller than the distance between adjacent points, only one transversally sectioned microvessel could occupy a given point. Microvessels transversally sectioned outside the points and those longitudinally or tangentially sectioned were excluded. Thus, a given microvessel was counted only once, even in the presence of several of its section planes. As nearly the entire section of each of three non-adjacent sections per sample was analysed, and as transversally sectioned microvessels hit the intersection points randomly, the method allowed objective counts. The microvessel area was then measured as the sum of points that hit microvessels, and expressed as mean percentage±1 s.d. for each section, sample and group of samples.

### Immunofluorescence and fluorescence *in situ* hybridisation analysis (FICTION)

In a preliminary set of experiments, endothelial cells were first identified by immunofluorescence using a rat anti-mouse CD34 (Dako, clone MEC 14.7) and a mouse anti-human CD31 (Dako, clone JC70A); a goat FITC-conjugated anti-rat (Dako) and an Alexa Fluor® rabbit anti-mouse (Invitrogen, Paisley, UK) antibody were used as secondary reagents.

FICTION (fluorescence immunophenotyping and interphase cytogenetics) experiments were performed on 4-*μ*m-thick paraffin sections of the tumour samples according to the previously described protocol (Martinez-Ramirez *et al*, 2004). Precisely, in a second set of experiments, immunofluorescence was performed either with the anti-mouse CD34 or the anti-human CD31, which were respectively developed with a goat FITC-conjugated anti-rat or with a rabbit FITC-conjugated anti-mouse antibody, from Dako, according to Weber-Matthiesen *et al* (1992). After immunostaining, which stained endothelial cells in green, fluorescence *in situ* hybridisation (FISH) was performed using either the TRITC-labelled centromeric probe specific for the human chromosome 1 (Qbiogene, Illkirch, Cedex, France) or a mouse Cot1-DNA (Invitrogen) labelled with Spectrum Orange deoxyuridine triphosphate using a nick translation kit according to the manufacturer's instructions (Vysis, Downers Grove, IL, USA). Afterwards, slides were washed and mounted in antifade solution with DAPI (Vectashield, Vector Burlingame, CA, USA). Images were captured using a Nikon Eclipse E1000 epifluorescence microscope (Nikon Corp., Tokyo, Japan) equipped with filter sets for DAPI (nuclei counterstaining), FITC (immunofluorescence signals) and TRITC (FISH signals).

### Terminal deoxynucleotidyl transferase-mediated dUTP nick end labelling assay

DNA cleavage was assessed by enzymatic end-labelling of DNA strand breaks using a commercial kit (*In Situ* Cell Death Detection Kit; Roche, Penzberg, Germany) according to the manufacturer's instructions. Briefly, deparaffinised slides with sections of *ACN/IFN-γ* or *ACN/neo* xenografts were washed in phosphate-buffered saline (PBS) and permeabilised with 0.1% Triton X-100 and 0.1% sodium citrate for 2 min at 4°C; after rinsing, slides were incubated with 50 *μ*l of terminal deoxynucleotidyl transferase (TdT)-mediated dUTP nick end labelling (TUNEL) reaction mixture, containing TdT- and FITC-labelled dUTP, in a humidified atmosphere for 1 h at 37°C in the dark. Afterwards, slides were rinsed and immunofluorescence was performed as described above with either anti-human CD31 or anti-mouse CD34, developed with a goat PE-conjugated anti-rat (Dako) and a rabbit PE-conjugated anti-mouse (Dako) antibody, respectively. Rinsed slides were then mounted in antifade solution with DAPI and images were captured as above with filter sets for DAPI (nuclei counterstaining), FITC (TUNEL) and TRITC (immunofluorescence). The percentages of apoptotic human and murine endothelial cells was calculated by dividing the TUNEL-positive CD31+ or CD34+ cells, respectively, by the total number of CD31+ or CD34+ cells counted in two slides for each assay. A Kruskal–Wallis test was then performed to assess statistical significance.

### Nitrate assay

NO production was evaluated as NO_2_ accumulation (Melillo *et al*, 1996). Briefly, cell-free supernatants of 48 h culture of *ACN/IFN-γ* or *ACN/neo* cells were incubated with the Griess reagent for 10 min at room temperature in triplicate and the absorbance measured in a 96-well plate reader (SPECTRAfluor Plus, TECAN, Grodig, Austria) at 550 nm. The concentration was determined through a sodium nitrite standard curve. The limit of sensitivity was 1.5 *μ*M.

### Chorioallantoic membrane assay

Fertilised White Leghorn chicken eggs (20 for each series) were incubated at 37°C at constant humidity. On the third incubation day, a square window was opened in the eggshell after removal of 2–3 ml of albumen to detach the developing CAM from the shell. The window was sealed with a glass and the eggs were returned to the incubator. On day 8, CAM were implanted, under sterile conditions within a laminar flow hood, with 1 mm^3^ sterilised gelatin sponges (Gelfoam; Upjohn Co., Kalamazoo, MI, USA) (Ribatti *et al*, 1997). Sponges were loaded with 1 *μ*l PBS or with 500 ng human recombinant FGF-2 (R&D Systems, Minneapolis, MN, USA) as negative and positive controls, respectively. Snap-frozen *ACN/neo* and *ACN/IFN-γ* tumours were minced in sterile RPMI 1640 to obtain 1–2 mm^3^ fragments, which were grafted onto the CAM of chick embryos on day 8, as previously described (Ribatti *et al*, 2002). Care was taken to select necrosis- and bleeding-free fragments. The CAM were examined daily until day 12 and photographed *in ovo* with a stereomicroscope equipped with an MC 63 Camera System (Zeiss, Oberkochen, Germany). On incubation day 12, when the angiogenic response peaked, blood vessels entering the implant within the focal plane of the CAM were recognised macroscopically, counted at × 50 magnification by two observers (DR and BN) in a double-blind fashion with a stereomicroscope and photographed. Mean values ±1 s.d. for vessel count were determined for each analysis. The CAM were also processed for light microscopy. Serial sections (8 *μ*m) were cut in a plane parallel to the surface of the CAM, stained with a 0.5% acqueous solution of toluidine blue (Merck, Darmstadt, Germany) and observed under a Leitz-Dialux 20 light photomicroscope (Leitz, Wetzlar, Germany). Some sections were also stained using the mAb anti-CD31 and the microvessel area inside the tumour xenografts was evaluated as described above.

### Statistics

Statistical significance of differences observed between the experimental (*ACN/IFN-γ*) and control (*ACN/neo*) groups was determined using the Student's *t-*test for unpaired data. Statistical significance of differences in apoptosis of human and murine endothelial cells in *ACN/IFN-γ* and *ACN/neo* xenografts was determined by means of the non-parametric Kruskal–Wallis test.

## RESULTS

### Histopathological features and microvascular area of ACN xenografts

**Figure 1:**
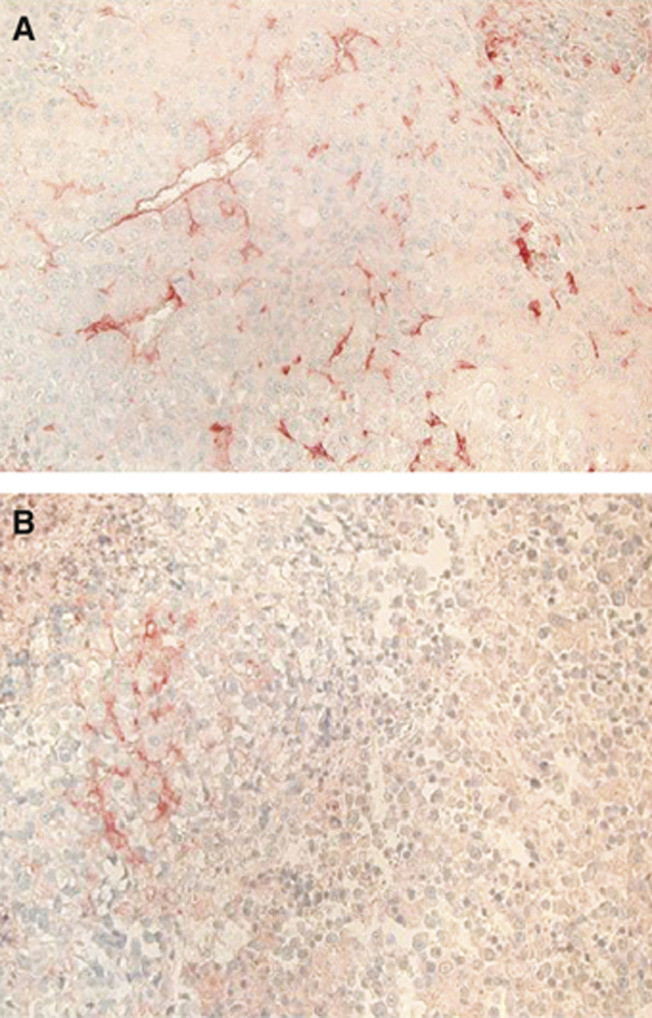
Immunohistochemical analysis of *ACN/neo* (**A**) and *ACN*/*IFN-γ* (**B**) tumours removed 14 days after injection. The microvessel density, determined by means of an anti-CD31 antibody recognising endothelial cells, was significantly (*P*<0.001) higher in *ACN/neo* than in *ACN*/*IFN-γ* tumours. Original magnification: × 160.

As opposed to parental and vector-transfected ACN xenografts showing numerous blood vessels and a well-defined vascularisation (Corrias *et al*, 1998), *ACN/IFN-γ* xenografts are characterised by extensive necrotic areas and focal basement membrane destruction (Airoldi *et al*, 2004). We thus evaluated the size of the microvessel area in the *ACN/IFN-γ* and *ACN/neo* xenografts. Tumours removed on day 14 p.i. and stained with anti-CD31, which selectively identifies microvessels and their long off shots reaching into the stroma, are shown in Figure 1A and B, respectively (microvessel density=3.5% for *ACN/neo vs* 11.2% for *ACN/IFN-γ* xenografts, *P*<0.001). Microvessel areas were significantly smaller in *ACN/IFN-γ* than in *ACN/neo* specimens at any time tested.

### Characterisation of tumour endothelium in ACN xenografts by FICTION

**Figure 2:**
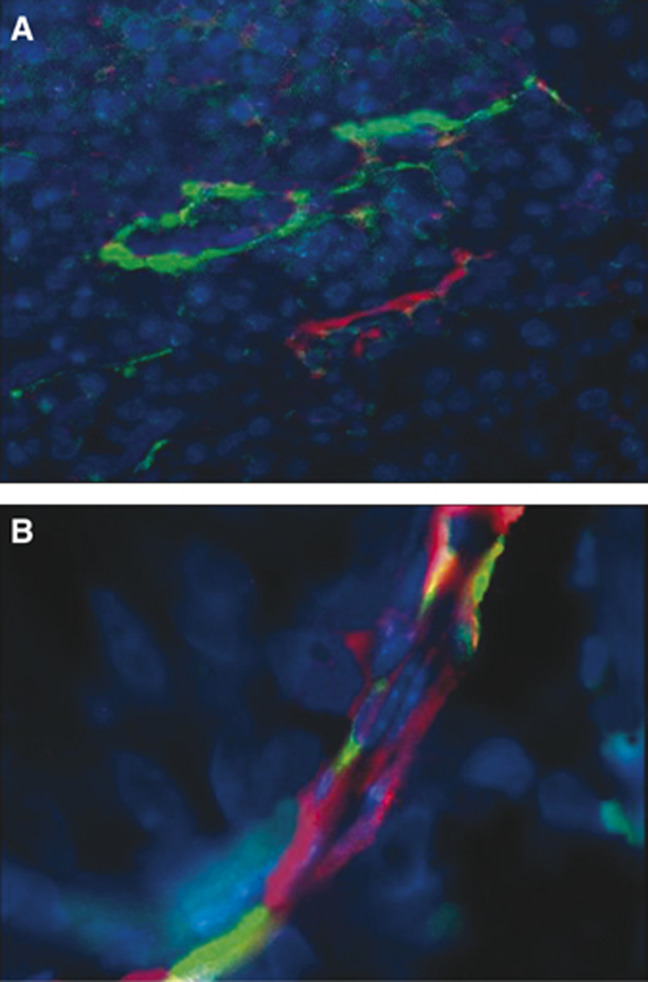
Immunofluorescence performed on *ACN/IFN-γ* paraffin-embedded sections. (**A**) Section simultaneously incubated with a rat anti-mouse CD34 and a mouse anti-human CD31 antibody developed, respectively, with a goat FITC-conjugated anti-rat and an Alexa Fluor® rabbit anti-mouse (human CD31 positive: red; murine CD34 positive: green). Original magnification: × 1000. (**B**) Section incubated as in (**A**) where a microvessel composed of both red and green cells is shown.

As microvascular endothelial cells may exhibit the same genetic aberrations as tumour cells (Gunsilius, 2003; Hida *et al*, 2004; Streubel *et al*, 2004), we first analysed whether the tumour vasculature in *ACN/neo* and *ACN/IFN-γ* xenografts contained tumour-derived endothelial cells. As shown in Figure 2A, microvessels of both human and murine origin were found in both xenografts. Quite interestingly, individual microvessels composed of both human and murine cells were observed (Figure 2B). Next, we applied the FICTION technique to confirm that the human endothelial cells were derived from the tumour cells. In fact, using this technique, which combines immunofluorescence and FISH (Weber-Matthiesen *et al*, 1992), it is possible to identify a given cell type, detected by mAb staining, on the basis of specific genetic profile.

**Figure 3:**
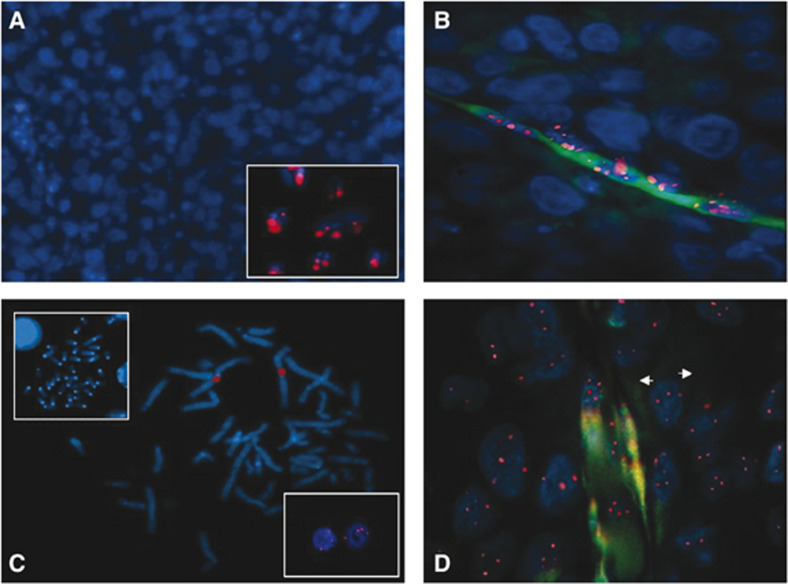
FICTION analysis of *ACN/IFN-γ* paraffin-embedded sections. (**A**) *ACN/IFN-γ* sections hybridised with the murine Cot-1 probe. The murine cell line TAP-1 hybridised with the Cot-1 probe is shown in the inset. (**B**) Identification of microvessel of mouse origin with anti-mouse CD34 (green) and a Cot-1 probe (red). (**C**) Human metaphase hybridised with the centromere-specific human chromosome 1 probe (red). Murine metaphase hybridised with the same probe is shown in the upper inset. ACN cells hybridised with the same probe are shown in the lower inset. (**D**) Identification of microvessels of human origin with anti-human CD31 (green) and the centromere-specific human chromosome 1 probe (red). Original magnification: (**A**) × 200, (**B**–**D**) × 1000.

Thus, after immunofluorescence performed using either anti-human CD31 or anti-mouse CD34 and FITC-conjugated secondary reagents, slides were hybridised to a mouse Cot1-DNA or to a centromere-specific human chromosome 1 probe that were labelled in red. ACN cells did not hybridise to the Cot-1 probe (Figure 3A), whereas murine cells showed large red fluorescent signals (Figure 3A, inset). The same hydridisation signals were evident in CD34-positive endothelial cells (Figure 3B), confirming their murine origin.

Conversely, the centromere-specific human chromosome 1 probe hybridised to human (Figure 3C) but not murine (Figure 3C, upper inset) metaphases. ACN cells showed several signals (Figure 3C, lower inset) indicative of chromosome 1 aneuploidy. After hybridisation with the centromere-specific human chromosome 1 probe, CD31-positive endothelial cells (Figure 3D) showed the same pattern as tumour cells, thereby demonstrating that endothelial cells of human origin were indeed derived from the human tumour cells.

Although the absolute number of murine microvessels was lower in *ACN/IFN-γ* xenografts than in *ACN/neo* xenografts, their percentages were similar in both tumours (70±4 *vs* 69±3, three serial sections for each xenograft), indicating that the ratio between murine and human vessels (approximately 2 : 1) was unaffected by IFN-*γ*.

### Interferon-*γ* released by transfected cells induced apoptosis of endothelial cells

**Figure 4:**
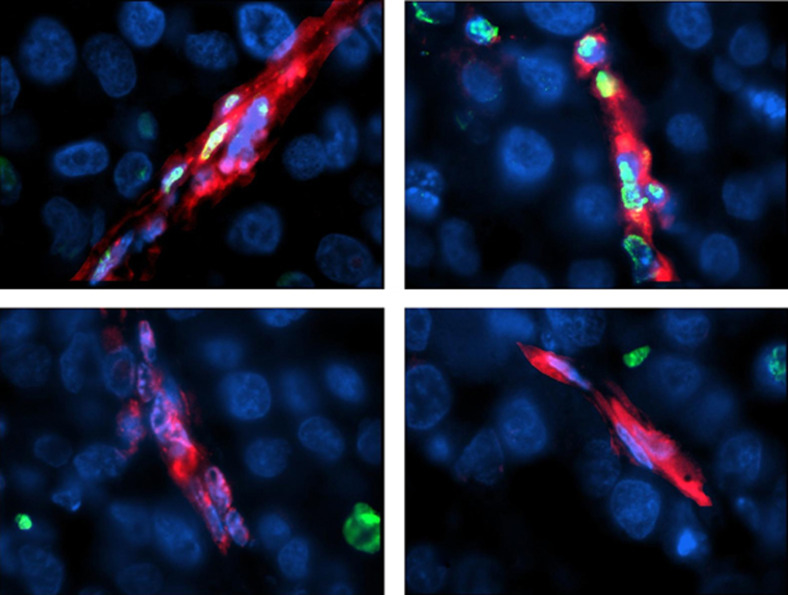
Immunofluorescence and TUNEL assay. *ACN*/*IFN-γ* (**A**, **B**) and *ACN/neo* (**C**, **D**) deparaffinized sections were subject to TUNEL assay (green) and then stained by immunofluorescence in red using either anti-murine CD34 (**A**, **C**) or anti-human CD31 (**B**, **D**) mAbs. Colocalisation of red and green (yellow) indicated apoptosis of endothelial cells. Original magnification: (**A**–**D**) × 1000.

In order to elucidate the mechanism(s) by which the released IFN-*γ* inhibited angiogenesis, the expression of the known antiangiogenic, IFN-*γ*-induced, human CXCL10 and CXCL9 proteins was analysed in both *ACN/IFN-γ* and *ACN/neo* xenografts. As neither protein was detected (data not shown), we checked whether apoptosis of endothelial cells occurred in the *ACN/IFN-γ* xenografts rather than the *ACN/neo* xenografts, by means of the TUNEL assay. Cells positive for TUNEL were then identified by immunofluorescence using either anti-murine CD34 or anti-human CD31 mAbs. Whereas in the *ACN/IFN-γ* xenografts, both murine and human endothelial cells were TUNEL-positive (75±3 and 52±3%, Figure 4A and B, respectively), no apoptosis of either murine- or human-derived endothelial cells was observed in *ACN/neo* xenografts (2±1 and 3±3%, Figure 4C and D, respectively). The difference in apoptotic cells between *ACN/IFN-γ* and *ACN/neo* xenografts was significant (*P*=0.002) regardless of the murine or human nature of the endothelial cells. In the *ACN/IFN-γ* xenograft, the proportion of TUNEL-positive murine endothelial cells was higher (*P*=0.002) than that of human-derived endothelial cells.

To investigate the possible mediator of the apoptotic signal affecting both murine and human endothelial cells, production of NO was measured in the supernatants from *ACN/IFN-γ* and *ACN/neo* cells cultured *in vitro*. The *ACN/IFN-γ* supernatants contained about 10 *μ*M NO, whereas no detectable NO was found in the *ACN/neo* supernatants (*P*<0.05).

### Inhibition of CAM vascularisation by sponges treated with *ACN/IFN-γ* tumours

**Figure 5:**
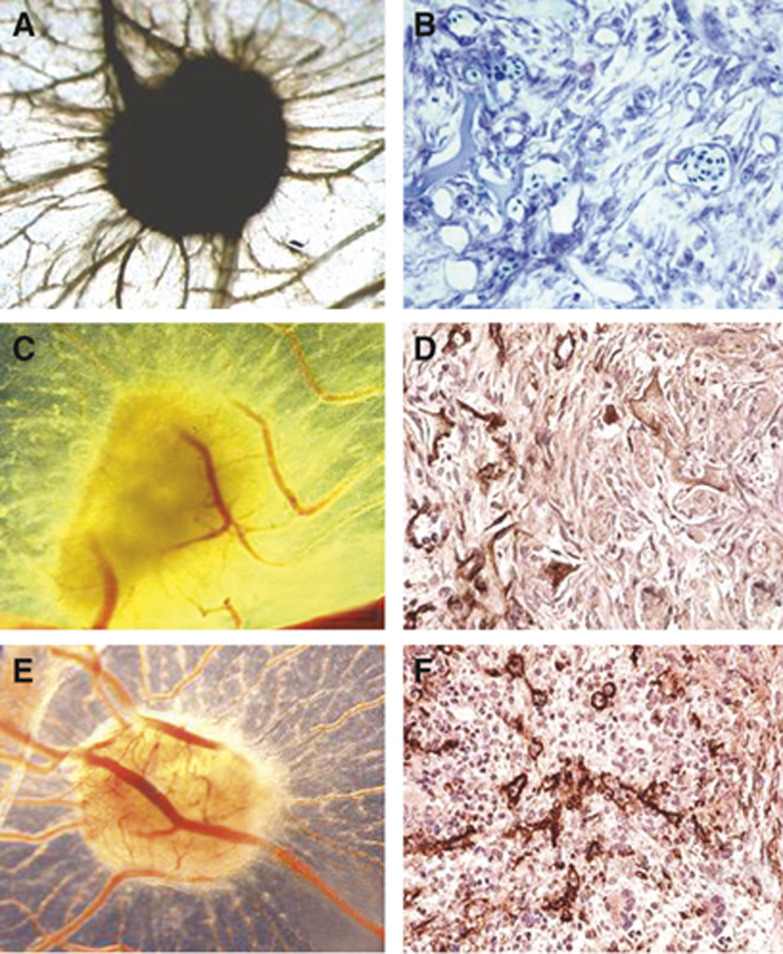
(**A**) A 12-day-old chick embryo CAM incubated on day 8 for 4 days with a gelatin sponge loaded with 500 ng hrFGF-2. Note numerous allantoic blood vessels with a radially arranged ‘spoked wheel’ pattern around the sponge. (**B**) Histological section of the sponge shown in (**A**). Note numerous small blood vessels among the sponge trabeculae intermingled with a collagenous matrix. (**C**) A 12-day-old CAM incubated on day 8 for 4 days with bioptic specimen of *ACN*/*IFN-γ* tumour xenograft, showing few vessels around the graft. (**D**) Immunohistochemical analysis of the xenograft, showing few CD31-positive blood vessels. (**E**) A 12-day-old CAM incubated on day 8 for 4 days with bioptic specimen of *ACN/neo* tumour xenograft, showing numerous blood vessels around the graft. (**F**) Immunohistochemical analysis of the xenograft, showing numerous CD31-positive blood vessels. The microvessel density, determined by means of an anti-CD31 antibody recognising endothelial cells, is significantly (*P*<0.001) lower in *ACN/IFN-γ* than in *ACN*/neo xenografts. Original magnification: (**A**, **C**, **E**) × 50; (**B**, **D**, **F**) × 400.

To further evaluate the *in vivo* angiostatic activity of human IFN-*γ* released by transfected cells, CAM vascularisation assays were performed (Ribatti *et al*, 1997). Gelatin sponges treated with hrFGF-2 (positive control) were surrounded by allantoic vessels that developed radially in a ‘spoked wheel’ pattern towards them (Figure 5A). Microscopically, a highly vascularised tissue was recognisable among the sponge trabeculae as newly formed blood vessels within an abundant network of collagen fibres (Figure 5B). When the sponges were loaded with PBS (negative control), physiological angiogenesis was observed in the form of some allantoic vessels partly arranged around the sponge (not shown). Microscopically, there were no blood vessels among the sponge trabeculae (not shown).

When the CAM assay was performed using *ACN/IFN-γ* or *ACN/neo* tumour xenografts, the number of vessels macroscopically counted around the implant was significantly lower in *ACN*/*IFN-γ* than in *ACN/neo* biopsies (15±3 *vs* 34±4, *P*<0.001, as shown in Figure 5C and E, respectively). Microscopically, the CAM area away from the implant was made up of a surface epithelium arising from the ectoderm (chorion), an intermediate mesenchyme containing arterious and venous vessels merging with a capillary network running under the chorion and a deep epithelium arising from the ectoderm (allantois). Tumour implants adhered to the chorion without invading the mesenchyme. The CAM vessels were arranged radially beneath the implants, and were less numerous in *ACN/IFN-γ* than in *ACN/neo* xenografts. A significant (*P*<0.001) decrease in microvessel area (1.2%; Figure 5D), evaluated as CD31-positive area, was detected in *ACN/IFN-γ* xenografts compared to *ACN/neo* grafts (5.8%; Figure 5F).

## DISCUSSION

Inhibitors of angiogenesis block any of the several steps in the angiogenic cascade, including proliferation and attachment of endothelial cells to the extracellular matrix proteins, as well as migration and invasion through the matrix (Bikfalvi and Bicknell, 2002). Here, we show that IFN-*γ* transfection of human NB cells exploited an antiangiogenic effect, measured by the lower microvessel density and the lower angiogenic potential in CAM assay of *ACN/IFN-γ* xenografts with respect to vector-transfected *ACN/neo* xenografts. Thus, human IFN-*γ* can affect NB tumour growth by inhibiting both tumour angiogenesis and proliferation (Airoldi *et al*, 2004). Two types of endothelial microvessels were detected in ACN xenografts, one being of murine origin and the other deriving from human tumour cells. Finally, in the *ACN/IFN-γ* xenografts, a high percentage of apoptotic murine and human endothelial cells were detected.

Transfection of human IFN-*γ* in other tumour cell types already suggested that IFN-*γ* inhibited angiogenesis. The acquired immunity against IFN-*γ*-transfected RT2 glioma cells was postulated to be primarily caused by the antiangiogenic activity of the secreted cytokine (Fathallah-Shaykh *et al*, 1998). Qin and Blankenstein (2000) and Qin *et al* (2003) showed that rejection of different tumours by CD8+ T cells was always preceded by inhibition of tumour-induced angiogenesis. Moreover, in some murine models, the antiangiogenic activity mediated by IL-12 and IL-18 was shown to be IFN-*γ* mediated (Voest *et al*, 1995; Cao *et al*, 1999), and inhibition of angiogenesis by IFN-*γ* was found to occur in colon carcinoma through transcriptional silencing of perlecan gene expression (Sharma and Iozzo, 1998).

The antiangiogenic effect observed in the *ACN/IFN-γ* xenografts was not mediated by the CXCL9 and CXCL10 proteins, a finding that contrasts with results from other tumour models (Strieter *et al*, 1995, Sgadari *et al*, 1996, Pertl *et al*, 2001) and with our previous observation that CXCL10 (IP-10) mRNA was induced in IFN-*γ*-transfected ACN cells (Airoldi *et al*, 2004). The antiangiogenic effect was mainly due to apoptosis of both murine- and tumour-derived endothelial cells, reflecting previous findings in IFN-*γ*-transfected brain tumour cells (Fathallah-Shaykh *et al*, 2000). The p38 MAPK/Stat1/IRF-1 pathway (Wang *et al*, 1999; Huang *et al*, 2002; Lee *et al*, 2005), cathepsin B (Li and Pober, 2005), Fas/FasL interaction (Li *et al*, 2002), integrin function (Ruegg *et al*, 1998) and NO production (Yamaoka *et al*, 2002; Vekemans *et al*, 2004; Lee *et al*, 2005) have been shown to drive IFN-*γ*-dependent apoptosis of endothelial cells. As not only human but also murine endothelial cells in *ACN/IFN-γ* xenografts were TUNEL-positive, neither the released human IFN-*γ* nor other species-specific mediators could be involved in mediating apoptosis in our model. Moreover, absence of FasL expression in the ACN and *ACN/IFN-γ* cells (not shown), and lack of infiltrating lymphocytes in the xenografts, excluded involvement of the Fas/FasL axis, at variance with other models (Sidky and Borden, 1987; Saiki *et al*, 1992). The demonstration that the *ACN/IFN-γ* cells produced NO strongly supported its role in driving the apoptotic signal to the endothelial cells. In this view, it is conceivable that a non-species-specific mediator, like NO, was responsible for inhibition of angiogenesis observed in other human IFN-transfected tumour cells growing in athymic mice (Hock *et al*, 1993; Fathallah-Shaykh *et al*, 2000; Ozawa *et al*, 2001; Izawa *et al*, 2002; Qin *et al*, 2002).

In *ACN/IFN-γ* xenografts, the proportion of apoptotic murine was higher than that of human endothelial cells, whereas their relative ratio remained unaltered (2 : 1). These findings support the hypothesis that the antiangiogenic effect of IFN-*γ* on human endothelial cells required other mechanisms besides apoptosis. Human IFN-*γ* inhibits endothelial migration and proliferation (Brouty-Boye and Zetter, 1980; Friesel *et al*, 1987; Tsuruoka *et al*, 1988; Albini *et al*, 2000) and has strong antiproliferative effects on NB tumour cells (Ponzoni *et al*, 1992; Airoldi *et al*, 2004), from which the human endothelial cells derived. Thus, the antiangiogenic effect was likely due to a combination of apoptosis and reduced proliferation. In this regard, it is of note that whereas apoptosis of tumour-derived endothelial cells was high, that of the tumour cells themselves was negligible, suggesting that transdifferentiation of ACN tumour cells into endothelial cells was accompanied by pathway changes.

The remarkable inhibition of angiogenesis by IFN-*γ*-transfected NB cells reported here, together with our preliminary observation that human recombinant IFN-*γ* reduced vessel growth induced by exogenous FGF-2 in the CAM assay by 80% (data not shown), lends additional support to the concept that angiogenesis inhibition is part of the general mechanism of action of IFN-*γ*. The possibility that this cytokine can find a place as an angiostatic adjuvant in the treatment of unresponsive NB, whose survival is poor (Maris and Matthay, 1999), warrants further investigation.

In conclusion, our study demonstrates an antiangiogenic effect of IFN-*γ* through the induction of apoptosis of both murine- and tumour-derived endothelial cells, likely mediated by NO production. Thus, should the contribution of tumour cell-derived microvessels be confirmed in human NB primary tumours, as our preliminary findings with MYCN amplified NB tumours seem to indicate (Pezzolo *et al*, unpublished), NB xenografts made up of mixed murine- and tumour-derived endothelial cells may prove useful for the testing of the therapeutic activity of antiangiogenic compounds.
